# Association of the interaction between occupational hazard factors and IL-1β gene polymorphism with cognitive function in electrolytic aluminum workers

**DOI:** 10.3389/fgene.2025.1591908

**Published:** 2025-06-25

**Authors:** Youxing Li, Yaqin Pang, Dongshun Chen, Feiyu Lu, Hongyan Tian, Fengni Qin, Kuntao Wei, Ahmad Razali Bin Ishak, Mohd Shukri Bin Mohd Aris, Guangzi Qi

**Affiliations:** ^1^ College of Public Health, Youjiang Medical University for Nationalities, Baise, Guangxi, China; ^2^ Faculty of Health Sciences, Universiti Teknologi MARA, Puncak Alam, Malaysia; ^3^ Key Laboratory of Research on Environment and Population Health in aluminum mining areas (Youjiang Medical University for Nationalities), Education Department of Guangxi Zhuang Autonomous Region, Baise, China; ^4^ Key Laboratory of Research on Environmental pollution and health risk assessment, Youjiang Medical University for Nationalities, Baise, Guangxi, China; ^5^ College of Medical Laboratory Medicine, Youjiang Medical University for Nationalities, Baise, Guangxi, China; ^6^ Occupational Health and Safety Risk Management (OHSeRM) Research Initiative Group, Universiti Teknologi MARA, Puncak Alam, Selangor, Malaysia

**Keywords:** occupational factors, aluminum dust, IL-1β, genetic polymorphism, cognitive function, interaction

## Abstract

**Background:**

Various occupational hazards in the electrolytic aluminum environment have been linked to cognitive decline. However, the interactive effects of these hazards and genetic factors on cognitive function remain unclear.

**Objective:**

This study aimed to identify the primary occupational hazards, examine their interaction with IL-1β gene polymorphisms in relation to cognitive function.

**Methods:**

A cross-sectional study was conducted in June 2024 at an electrolytic aluminum company in China, involving 478 male workers. Cognitive function was assessed using the Montreal Cognitive Assessment. Calculate the cumulative exposure dose of harmful factors such as aluminum dust. Additionally, IL-1β gene polymorphisms (rs1143627, rs1143643, rs16944, rs3917356) and serum protein levels were analyzed. The associations between environmental exposure, genetic factors, and cognitive function were examined using multivariate stepwise linear regression, restricted cubic splines, generalized linear models, and hierarchical analysis. Covariance analysis and independent sample t-tests were employed to assess the potential mediating effect of peripheral blood IL-1β levels.

**Results:**

Cumulative exposure to aluminum dust was significantly associated with cognitive decline (β = −0.18, 95% CI: 0.27, −0.10), and the relationship was linear. Compared to the wild genotype, individuals carrying rs1143627 G/G, rs1143643 C/C, and rs16944 A/A exhibited significantly lower cognitive scores (*P* < 0.01), whereas rs3917356 C/T and T/T conferred a protective effect (*P* < 0.01). The model was adjusted for age, body mass index, and cumulative aluminum dust exposure. The genetic effect associated with IL-1β was more pronounced in individuals with high aluminum exposure (>2.37 mg/m^3^ × year). IL-1β serum protein levels showed no significant association with cognitive function (*P* > 0.05).

**Conclusion:**

Cumulative exposure to aluminum dust is a key risk factor for cognitive decline. IL-1β polymorphisms influence susceptibility, with the effect becoming more pronounced under high aluminum exposure. However, peripheral blood IL-1β levels do not mediate this association with cognitive decline.

## 1 Introduction

With ongoing industrialization and increasing awareness of occupational health, regulatory agencies continuously update and refine workplace exposure limits for hazardous factors to minimize their impact on workers’ health (Guo et al., 2024; Veer and Kumar, 2024). However, despite stringent regulatory frameworks, exposure concentrations of hazardous substances in occupational environments—such as those in the metal smelting industry—remain significantly higher than those encountered in everyday settings (Mozaffari et al., 2023; Silver et al., 2021). Research indicates that workers exposed chronically to physical and chemical hazards are at heightened risk for various occupational diseases, with cognitive dysfunction being one of the more insidious and often overlooked health impairments (Tharwani et al., 2024; Mohammed et al., 2020). This issue is particularly pronounced among aluminum workers (Meng et al., 2019). While most affected workers exhibit only mild cognitive impairment (MCI), studies suggest that approximately 8%–13% of individuals with MCI may progress to Alzheimer’s disease (AD) (McGirr et al., 2022; Kandimalla et al., 2016). Such cognitive decline not only threatens individual quality of life but also raises concerns regarding workplace safety and productivity. Given these risks, it is essential to identify the key occupational hazards contributing to cognitive decline and to screen high-risk individuals. Establishing effective prevention and intervention strategies will be crucial in mitigating the long-term health and occupational safety implications of these exposures.

Current research has established a strong association between aluminum and its compounds and the development of neurodegenerative diseases (Li et al., 2024; Zhang F. et al., 2024). While aluminum intake in daily life is relatively low, occupational exposure—particularly in the aluminum electrolysis industry—significantly increases the body’s aluminum burden. Elevated levels of aluminum exposure, as indicated by increased blood or urine aluminum concentrations, have been positively correlated with a higher incidence of cognitive dysfunction (Shang et al., 2021; Letzel et al., 2000). In these studies, demographic and behavioral characteristics such as age, gender, education, smoking, and alcohol consumption are commonly included as covariates in analytical models for adjustment. However, limited attention has been given to the influence of factors other than aluminum exposure on cognitive function (Lu, 2018). The aluminum electrolysis environment exposes workers to a range of harmful occupational factors, including high temperatures, noise, strong magnetic fields, sulfides, carbon oxides, nitrogen oxides, fluorides, and other physical and chemical hazards, all of which may have adverse effects on cognitive function (Yin et al., 2024; Modenese and Gobba, 2021; Meo et al., 2024). Furthermore, It is worth exploring whether these occupational hazards have a threshold effect.

In addition to occupational hazards, genetic susceptibility is one of the most critical factors influencing cognitive function. Research has demonstrated that neuroinflammation plays a pivotal role in the development of MCI and AD (Nordengen et al., 2019; Schuitemaker et al., 2009). Current studies suggest that, upon stimulation, microglia in the hippocampus activate the intracellular transcription factor NF-κB, leading to an upregulation of precursor interleukin-1β (pro-IL-1β) expression. Concurrently, activation of the NLRP3-caspase-1 inflammasome facilitates the maturation of IL-1β through proteolytic cleavage, ultimately inducing neuronal damage and contributing to cognitive dysfunction (Chen and Yu, 2023; Chesnokova et al., 2016). Notably, in real-world occupational settings, we observed substantial variations in the degree of cognitive impairment among workers exposed to identical environmental conditions. This observation suggests that genetic variants in key molecular pathways may significantly influence the onset and progression of cognitive impairmen. Based on this, we hypothesize that IL-1β genetic polymorphisms may modulate individual susceptibility to cognitive dysfunction.

To test this hypothesis and explore the interaction between IL-1β genetic polymorphisms and occupational hazardous factors in relation to cognitive function, a research study was conducted in a large aluminum electrolysis company. Individual cumulative exposures were quantified based on job type, years of work experience, and the level of exposure to hazardous factors. Simultaneously, four SNP loci of the IL-1β gene were genotyped in each participant, and their associations with cognitive function were analyzed. Additionally, IL-1β serum protein expression levels were measured to assess whether genetic variants and occupational hazards influence peripheral immune responses and, consequently, cognitive function.

## 2 Methods

### 2.1 Participants

A cross-sectional study was conducted, recruiting 478 male workers in June 2024 from a large electrolytic aluminum producer in Baise City, Guangxi Province. Inclusion criteria. The inclusion criteria: (a) Employed for at least 1 year in the company; (b) Adult males aged 18 years or olde. Exclusion criteria: (a) Diagnosed with cognitive impairment or severe chronic diseases; (b) Had first-degree relatives with Alzheimer’s disease; (c) Long-term use of aluminum-containing drugs or psychotropic medications; (d) Experienced major trauma within the last 2 months; (e) Had severe visual or auditory impairments or exhibited extreme uncooperative behavior. Ethical approval for this study was obtained from the Ethics Committee of Youjiang Medical University for Nationalities (2023070601) and the Ethics Committee of Universiti Teknologi MARA (REC/12/2023-PG/FB/29). All participants provided written informed consent before undergoing blood sample collection and cognitive function assessment.

### 2.2 Sample size estimation

Refer to the sample size calculation formula for cross-sectional studies:

$$N=\frac{Z_{1-\alpha /2}^{2}\times p\;\left(1-p\right)}{d^{2}}$$

In the formula, N represents the required sample size, Z_1-α/2_ is the critical value from the standard normal distribution, *p* refers to the expected prevalence of the disease in the target population, and *d* is the allowable margin of error. According to previous studies, the prevalence of cognitive impairment among aluminium workers is approximately 30% (Wang et al., 2020); therefore, *p* was set to 0.30. With a significance level of α = 0.05, the corresponding Z_1-α/2_ is 1.96, and *d* was set at 0.05, a commonly used value in epidemiological research. Substituting these values into the formula results in a required sample size of 323 participants. In this study, a total of 478 individuals were included, indicating that the sample size was sufficient.

### 2.3 Data collection

Demographic information was collected, including age, body mass index (BMI), marital status (living alone/cohabiting), ethnicity (Han Chinese/ethnic minorities), income level (<3,000 CNY, 3,000–5,000 CNY, >5,000 CNY), and education level (junior high school and below/High school and above). Additionally, data on smoking (at least one cigarette per day for ≥6 months; no) and drinking (yes: drinking alcohol at least once a week for ≥6 months; No) were recorded (Xiao et al., 2021). Cognitive function was assessed using the Montreal Cognitive Assessment (MoCA). In accordance with standard scoring criteria, participants with ≤12 years of education received an additional one-point adjustment, with a maximum total score of 30. Lower MoCA scores indicate poorer cognitive functioning.

### 2.4 Detection of occupational hazardous factors

Data on occupational hazardous factors were primarily obtained from the enterprise’s occupational disease hazard detection report. All detection procedures were conducted in strict accordance with the National Occupational Health Standard (GBZ/T). The detected occupational hazardous factors included high temperature, noise, magnetic fields, aluminum dust, nitrogen oxides (NO_x_), sulfur dioxide (SO_2_), carbon monoxide (CO), fluoride, and manganese oxide. For concentration values below the Limit of Detection (LOD), half of the lowest detection limit (LOD/2) was substituted. All test results were expressed as the 8-hour time-weighted average (8h-TWA), and the Cumulative Exposure Dose (CED) for each worker was calculated by multiplying the 8h-TWA by the number of years of service.

The calculation formula for CED is as follows: CED = ∑(Ci × Ti),where: CED represents the cumulative exposure dose (unit: concentration × year), Ci is the 8h-TWA for the *i*th time period, Ti is the exposure duration in the *i*th time period.

### 2.5 IL-1β SNP selection, genotyping and serum level determination

The selection of IL-1β SNPs was based on human genetic variation data from the National Center for Biotechnology Information (NCBI) database. The screening criterion required a minor allele frequency (MAF) greater than 5%. Four SNPs were selected: rs1143627, rs1143643, rs16944, and rs3917356. Previous studies have suggested that these loci may be associated with cognitive function (Zhuang et al., 2012; Huang et al., 2024; Stacey et al., 2017). Genotyping was conducted using the improved Multiplex Ligation Detection Reaction (iMLDR) technique. Serum IL-1β protein levels were measured using a commercial enzyme-linked immunosorbent assay (ELISA) kit (Andi Gene Biotechnology Co., Guangdong, China).

### 2.6 Statistical analysis

Age and BMI were expressed as mean ± standard deviation (Mean ± SD), while the accumulation of occupational hazardous factors was described using the median (25th, 75th percentiles). Categorical variables, including marital status, ethnicity, income, education, smoking status, and drinking status, were presented as counts and percentages (n, %).

Multivariable Stepwise Linear Regression (MSLR) was performed to evaluate the relationship between occupational hazards and cognitive function and to identify major influencing factors. Subsequently, dose-response curves for occupational hazards and cognitive function were fitted using Restricted Cubic Spline (RCS) to explore potential threshold effects.

A Generalized Linear Model (GLM) was used to assess the association between IL-1β gene polymorphisms and cognitive function. To further investigate the Gene-Environment Interaction (GENI), participants were stratified based on the threshold levels of major occupational hazards. The relationship between IL-1β gene polymorphisms and cognitive function was then re-evaluated using GLM.

A two-independent-samples t-test was used to compare differences in IL-1β serum protein expression levels between groups exposed to major occupational hazards. Additionally, general linear regression was applied to examine the association between IL-1β serum protein expression levels and cognitive function, further exploring the role of peripheral blood IL-1β in cognitive function.

All statistical analyses were performed using IBM SPSS Statistics 22.0, with a two-sided P-value <0.05 considered statistically significant.

## 3 Results

### 3.1 General characteristics of participants and occupational exposure

Table 1 presents the general characteristics of the 478 aluminum workers included in this study, along with their cumulative exposure levels to occupational hazardous factors. The mean age of participants was 39.45 years, and the mean BMI was 24.86. The majority of workers (85.8%) lived with a partner. Ethnic minorities accounted for the largest proportion of participants (66.1%). In terms of income level, most workers earned a monthly salary of 3,000–5,000 CNY (64%). Regarding educational attainment, 72.8% had at least a high school education. Concerning health behaviors, the prevalence of smoking was high (54.4%), while the proportion of alcohol consumption was relatively low (35.4%). Regarding occupational exposure factors, various occupational hazards exhibited some degree of cumulative exposure, indicating the long-term presence of potentially harmful environmental conditions in the workplace.

**TABLE 1:** General and occupational exposure characteristics of aluminum workers (n = 478).

Characteristics	Mean ± SD, n (%), median (25th, 75th)
General characteristics	
Age,mean ± SD e (years)	39.45 ± 6.90
BMI,mean ± SD (kg/m3)	24.86 ± 3.46
Marital status	
Living alone	68(14.2)
Cohabiting	410(85.8)
Ethnicity	
Han Chinese	162(33.9)
Ethnic minorities	316(66.1)
Income	
<3,000(CNY)	116(24.3)
3,000–5,000(CNY)	306(64)
>5,000(CNY)	56 (11.7)
Education level	
Junior high school and below	130 (27.2)
High school and above	348 (72.8)
Smoking	
No	218 (45.6)
Yes	260 (54.4)
Drinking	
No	309 (64.6)
Yes	169 (35.4)
Occupational Exposure	
High temperature^(oC^ × year^)^	318.75 (146.36, 468.19)
Noise (dB(A) × year)	921.38 (374.74, 1240.15)
Magnetic field (Tesla × year)	0.06 (0.00, 0.22)
Aluminum dust(mg/m^3^ × year)	2.37 (1.49, 6.36)
NO_X_ (mg/m^3^ × year)	0.35 (0.17, 0.57)
SO_2_ (mg/m^3^ × year)	1.72 (1.00, 3.67)
CO(mg/m^3^ × year)	1.45 (0.65, 2.15)
Fluoride(mg/m^3^ × year)	0.05 (0.03, 0.14)
Manganese oxide (mg/m^3^ × year))	0.02 (0.01, 0.02)

Abbreviations: SD: standard deviation; CNY, Chinese yuan. dB(A): A-weighted decibel.

### 3.2 Multiple stepwise linear regression analysis of cognitive functioning

Given the presence of multiple occupational hazards in the work environment and the strong correlations or covariances among these factors, this study employed multiple stepwise linear regression analysis to investigate the associations between occupational hazards and cognitive function among aluminum workers. The four factors (age, BMI, noise, and aluminum dust) were automatically selected and retained in the final model. As presented in Table 2, age[β (95% CI): 0.08(-0.13, −0.04)], BMI [β (95% CI): 0.08(-0.15, −0.01)] and aluminum dust [β (95% CI): 0.18(-0.27, −0.10)] were significantly negatively associated with cognitive function scores. Notably, noise exposure [β (95% CI): 0.001(0.001, 0.002)] exhibited a positive association with cognitive function scores. However, noise was not statistically associated with cognitive function in the one-way analyses, and it was statistically associated in the multifactorial analyses but the regression coefficients had small β-values and may have been influenced by other factors, and thus could be considered excluded in the final model.

**TABLE 2:** Results of the multivariate stepwise linear regression analysis of cognitive functions.

Variables	β(95%CI)^a^	*P*-value^a^	β(95%CI)^b^	*P*-value^b^
Age	−0.07(−0.11,-0.04)	<0.001	−0.08(−0.13,-0.04)	<0.001
BMI	−0.09(−0.16, −0.02)	0.028	−0.08(−0.15, −0.01)	0.036
Noise	<0.001(−0.001, 0.000)	<0.785	0.001(0.001, 0.002)	<0.001
Aluminum dust	−0.15(−0.23, −0.08)	<0.001	−0.18(−0.27, −0.10)	<0.001

Note:

^a^ one-way analyses.

^b^ multivariate stepwise linear regression analysis.

### 3.3 Dose-response relationship fitting for cognitive function

**FIGURE 1:**
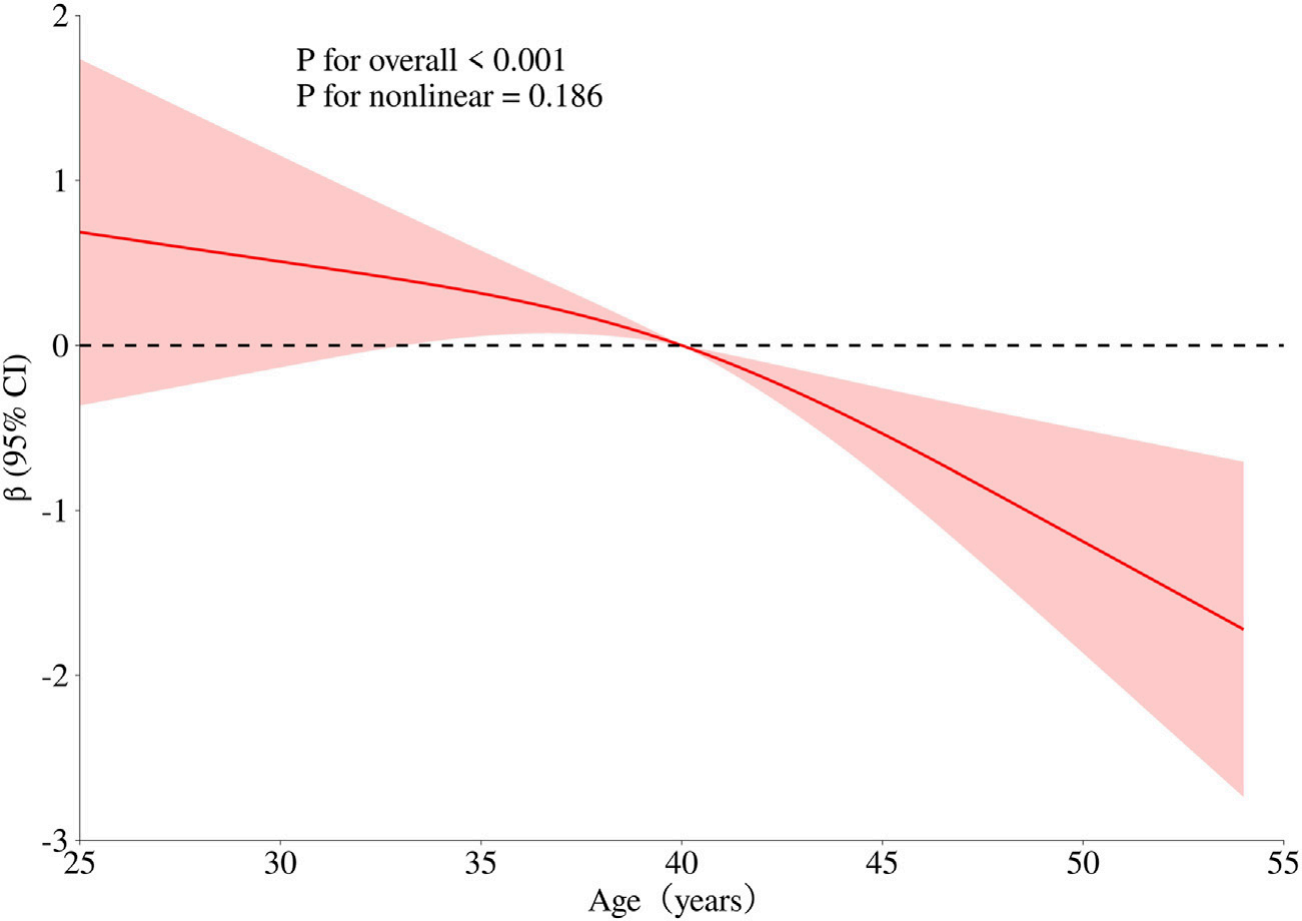
Dose-response relationship between age and cognitive function. Model adjusted for BMI, cumulative noise exposure and cumulative aluminum dust exposure.

**FIGURE 2:**
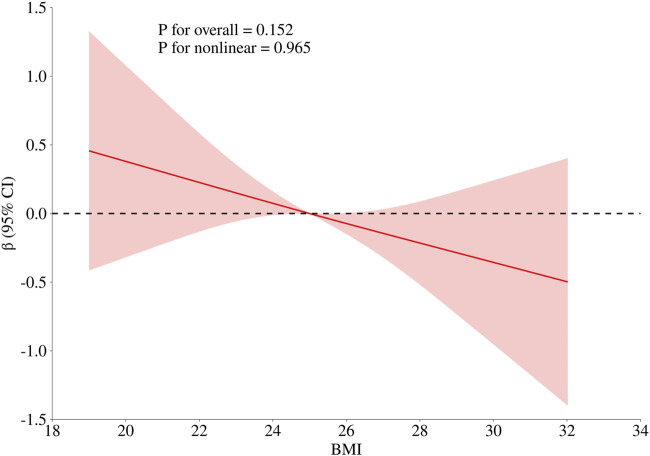
Dose-response relationship between BMI and cognitive function. The model was adjusted for age, cumulative noise exposure and cumulative aluminum dust exposure.

**FIGURE 3:**
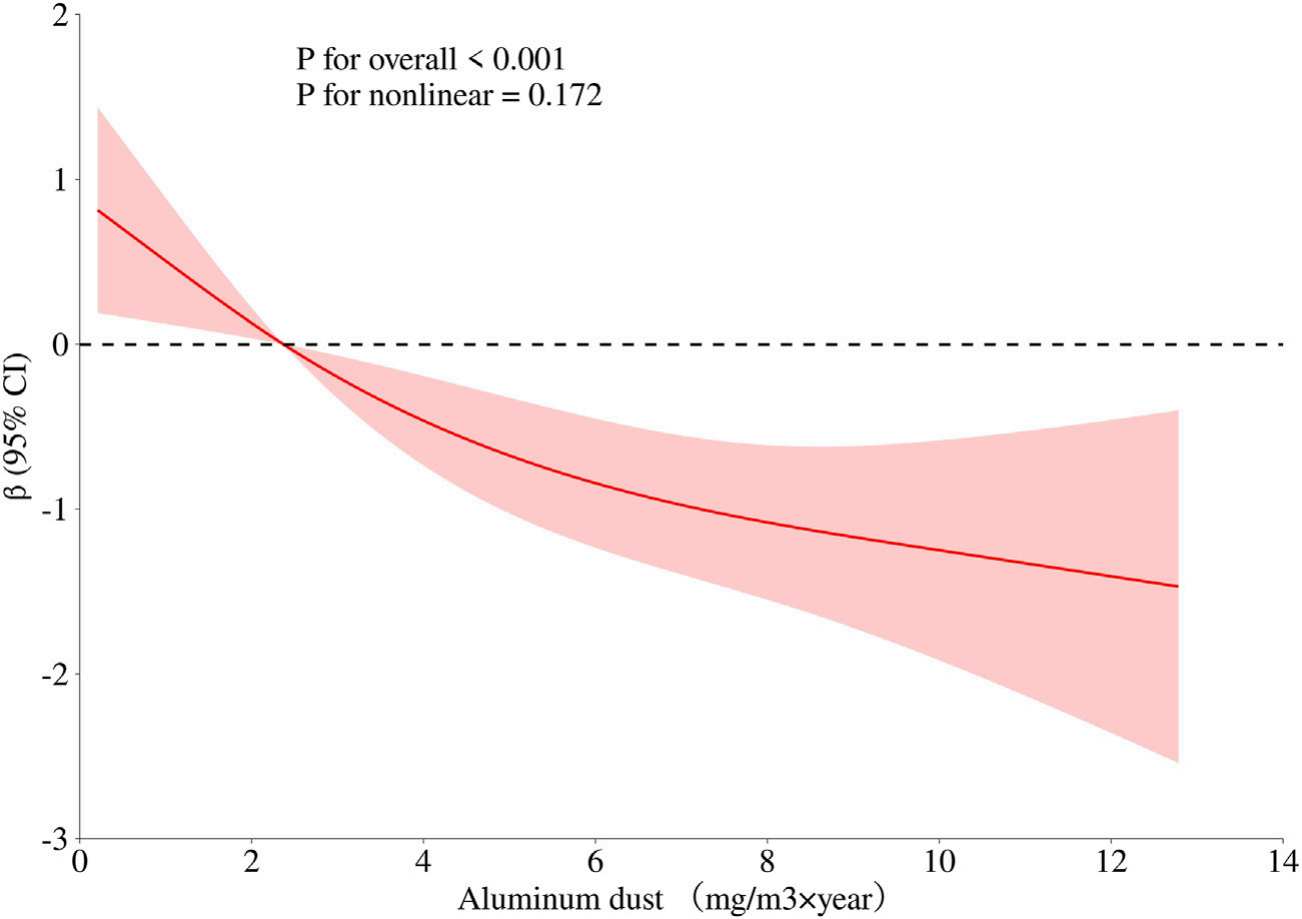
Dose-response relationship between cumulative aluminum dust exposure and cognitive function. Model adjusted for age, BMI and cumulative noise exposure.

To obtain a more comprehensive understanding of the dose-response relationship between age, BMI, cumulative aluminum dust exposure, and cognitive function, this study employed the RCS method to fit the dose-response curve. The number of spline knots was automatically selected based on the minimum Akaike Information Criterion (AIC) to determine the optimal model. The results are illustrated in Figures 1–3. Age exhibited a negative linear association with cognitive function scores (P overall <0.001, P nonlinear = 0.186). BMI was not significantly associated with cognitive function scores (*P* overall = 0.152, *P* nonlinear = 0.965). Cumulative aluminum dust exposure was negatively and linearly correlated with cognitive function scores (*P* overall <0.001, *P* nonlinear = 0.172), indicating the absence of a threshold effect. The 50th percentile of exposure was 2.37 mg/m^3^× year.

### 3.4 Analysis of the correlation between IL-1β gene polymorphisms and cognitive function

This study examined the association between four IL-1β gene SNPs (rs1143627, rs1143643, rs16944, rs3917356) and cognitive function scores. The results are presented in Table 3. In the adjusted model, compared to the wild-type genotype, individuals carrying the rs1143627G/G [β (95% CI): 1.22 (−1.94, −0.52)], rs1143643C/C [β (95% CI): 0.95 (−1.68, −0.23)] and rs16944 A/A [β (95% CI): 1.12 (−1.84, −0.39)] exhibited significantly lower cognitive function scores. These findings suggest that individuals carrying these mutant genotypes may be at a higher risk of cognitive decline. In contrast, individuals carrying the rs3917356C/T [β (95% CI): 0.88 (0.28, 1.47)] and T/T [β (95% CI): 1.27 (0.54, 1.99)] demonstrated higher cognitive function scores, indicating that these genotypes may exert a protective effect against cognitive decline.

**TABLE 3:** Association between IL-1β SNPs and cognitive function.

SNPs	n	MoCA ± SD	β (95% CI)^a^	*P-*value	β (95% CI)^b^	*P-*value
rs1143627						
A/A	126	26.17 ± 0.21	1		1	
G/A	235	25.63 ± 0.19	−0.54 (−1.17, 0.09)	0.093	−0.48 (−1.09, 0.13)	0.122
G/G	117	24.97 ± 0.32	**−1.19 (-1.92, -0.46)**	**0.001**	**−1.22 (−1.94, −0.52)**	**0.001**
rs1143643						
T/T	118	25.80 ± 0.24	1		1	
C/T	244	25.86 ± 0.18	0.06 (−0.58, 0.70)	0.854	0.01 (−0.62, 0.64)	0.975
C/C	116	24.91 ± 0.32	**−0.89 (−1.64, −0.15)**	**0.019**	**−0.95 (−1.68, −0.23)**	**0.010**
rs16944						
G/G	130	26.16 ± 0.21	1		1	
G/A	240	25.55 ± 0.19	−0.61 (−1.23, 0.008)	0.053	−0.58 (−1.19, 0.03)	0.059
A/A	108	25.08 ± 0.33	**−1.08 (−1.82, −0.34)**	**0.004**	**−1.12 (−1.84, −0.39)**	**0.002**
rs3917356						
C/C	139	24.97 ± 0.28			1	
C/T	239	25.73 ± 0.18	**0.76 (0.16, 1.37)**	**0.014**	**0.88 (0.28, 1.47)**	**0.001**
T/T	100	26.21 ± 0.24	**1.24 (0.50, 1.98)**	**0.001**	**1.27 (0.54, 1.99)**	**0.004**

Note:

^a^ Unadjusted model.

^b^ The model adjusted for age, BMI, and cumulative exposure to aluminum dust.

Bold values indicate statistically significant differences compared to the reference genotype.

### 3.5 Stratified analysis of the association between IL-1β gene polymorphisms and cognitive function

To further investigate the interaction between IL-1β gene polymorphisms and aluminum dust exposure, we performed a stratified analysis according to the median cumulative exposure to aluminum dust (2.37 mg/m^3^ × year). The results, presented in Table 4, In the low exposure group (≤2.37 mg/m^3^ × year), individuals carrying rs1143627 G/G exhibited lower cognitive function scores [β (95% CI): 0.92 (−1.83, −0.01)], while those carrying rs3917356 T/T had higher cognitive function scores [β (95% CI): 0.99 (0.05, 1.92)]. In the high exposure group (>2.37 mg/m^3^ × year), carrying rs1143627G/G [β (95% CI): 1.65(-2.71, −0.59)], rs1143643C/C[β (95% CI): 1.17(-2.26, −0.08)], and rs16944 A/A [β (95% CI): 1.46(-2.53, −0.38)] was associated with lower cognitive function scores. Conversely, carrying rs3917356C/T [β (95% CI): 1.31(0.45, 2.16))] and T/T [β (95% CI): 1.59(0.53, 2.66)] was associated with higher cognitive function scores. Notably, the interaction between each SNP locus and aluminum exposure was not statistically significant. However, the trend in the data suggests that the effect values (β) of risk genotypes increased in the high exposure group, indicating a potential environmental modification. This finding implies that aluminum dust exposure may amplify the impact of genetic variation on cognitive function, though further studies with larger samples are needed for validation.

**TABLE 4:** Stratified analysis of IL-1β SNPs and cognitive function (based on cumulative exposure to aluminum dust).

SNPs	≤2.37 C β (95%CI)	*P*-value	>2.37(mg/m^3^× year) β (95%CI)	*P-*value	*P*_*interation*_
rs1143627					0.551
A/A	1		1		
G/A	−0.43(−1.23, 0.38)	0.300	−0.43(−1.31, 0.46)	0.343	
G/G	**−0.92(−1.83, −0.01)**	**0.046**	**−1.65(−2.71, −0.59)**	**0.002**	
rs1143643					0.727
T/T	1		1		
C/T	0.09(−0.73, 0.91)	0.832	0.11(−0.80, 1.01)	0.813	
C/C	−0.77(−1.69, 0.15)	0.101	**−1.17(−2.26, −0.08)**	**0.036**	
rs16944					0.733
G/G	1		1		
G/A	−0.51(−1.31, 0.28)	0.206	−0.53(−1.40, 0.34)	0.231	
A/A	−0.89(−1.81, 0.04)	0.059	**−1.46(−2.53, −0.38)**	**0.008**	
rs3917356					0.613
C/C	1		1		
C/T	0.59(−0.19, 1.37)	0.136	**1.31(0.45, 2.16)**	**0.003**	
T/T	**0.99(0.05, 1.92)**	**0.039**	**1.59(0.53, 2.66)**	**0.003**	

Note: The model has been adjusted for age and BMI.

Bold values indicate statistically significant differences compared to the reference genotype.

### 3.6 Comparison of IL-1β serum protein expression levels across different IL-1β SNPs and aluminum dust exposure groups

**FIGURE 4:**
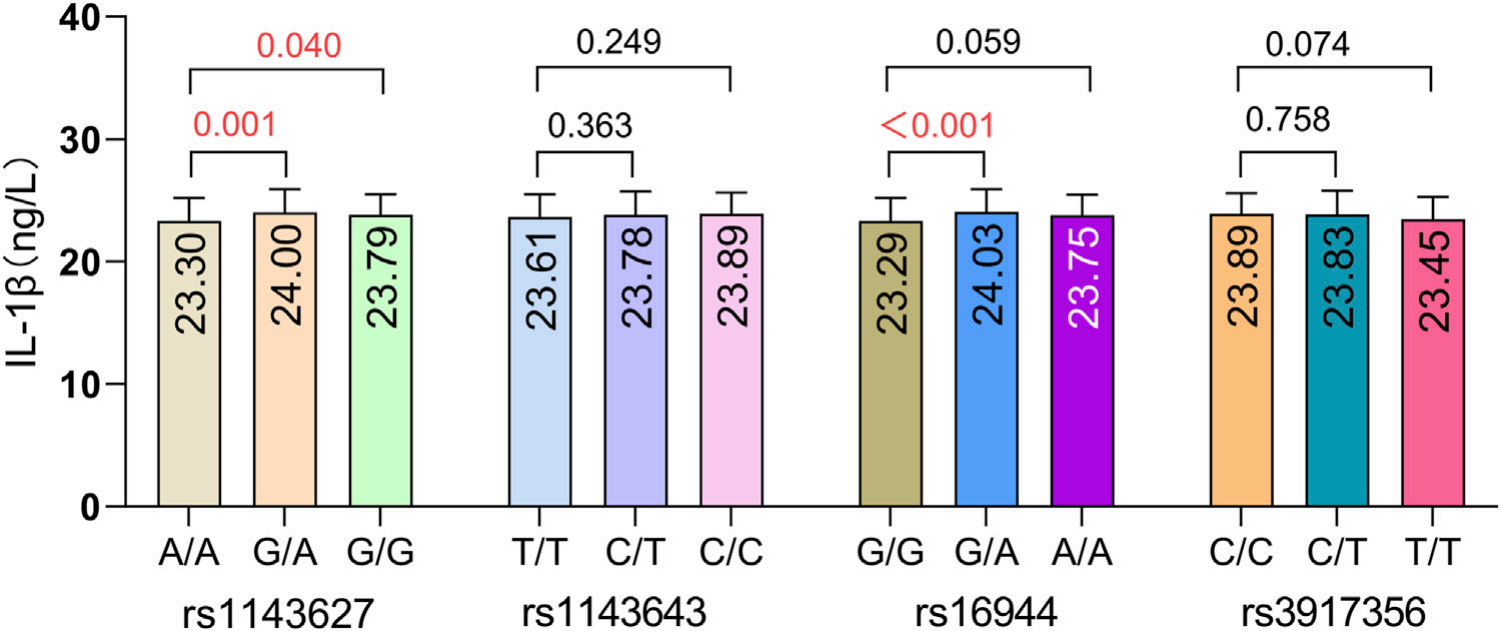
Comparison of IL-1β serum protein expression levels across different rs1143627, rs1143643, rs16944, and rs3917356 genotypes. The model was adjusted for age, BMI, and cumulative aluminum dust exposure.

**FIGURE 5:**
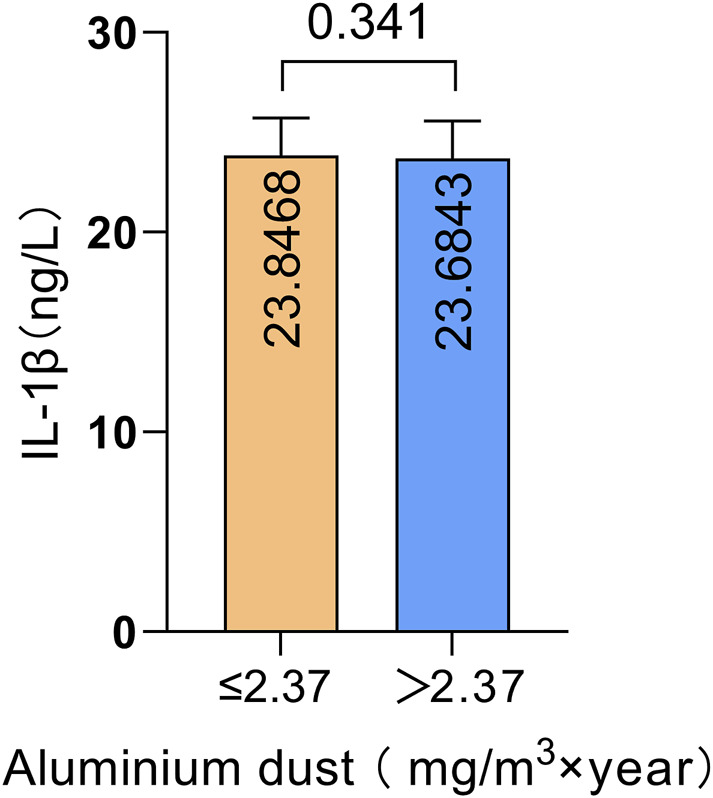
Comparison of IL-1β serum protein expression levels between groups stratified by cumulative aluminum dust exposure.

We compared IL-1β serum protein expression levels across different genotypes at four IL-1β SNPs. The results are illustrated in Figure 4. Compared to the wild-type genotype, individuals carrying the rs1143627G/A and G/G genotypes exhibited significantly higher IL-1β serum protein expression levels than those with the A/A genotype (P = 0.001, P = 0.040). Additionally, individuals carrying the rs16944 G/A genotype had significantly higher IL-1β serum protein expression levels compared to those with the G/G genotype (*P* < 0.001). These findings suggest that genetic polymorphisms at these two loci (rs1143627 and rs16944) may influence IL-1β protein expression levels in peripheral blood. The effect of aluminum dust exposure on IL-1β serum protein expression was analyzed separately (Figure 5). There was no significant difference in IL-1β serum protein levels between groups stratified by cumulative aluminum dust exposure (*P* = 0.341). This suggests that the damaging effect of aluminum on cognitive function may not be mediated through peripheral inflammation, although further research is needed to confirm this hypothesis.

### 3.7 Correlation analysis of IL-1β serum protein expression levels and cognitive function

The results, presented in Table 5, indicate that IL-1β serum protein expression levels were not significantly correlated with cognitive function scores, regardless of whether other influencing factors were adjusted. These findings further suggest that peripheral inflammation may not play a mediating role in cognitive decline.

**TABLE 5:** Association between IL-1β serum protein expression levels and cognitive function.

Variable	β(95%CI)^a^	*P*-value	β(95%CI)^b^	*P*-value
IL-β (ng/L)	−0.004(−0.145, 0.137)	0.957	−0.002(−0.139, −0.136)	0.981

Note:

^a^ Unadjusted model.

^b^ The model adjusted for age, BMI, and cumulative exposure to aluminum dust.

## 4 Discussion

In this study, we evaluated the association between cumulative exposure to occupational hazardous factors in the electrolytic aluminum environment and cognitive function. Additionally, we explored whether individual genetic susceptibility, gene-environment interactions, and the peripheral pro-inflammatory factor IL-1β play a mediating role in cognitive decline. The results demonstrated that cumulative aluminum dust exposure (CED) is a major factor contributing to cognitive decline. Furthermore, individuals carrying the IL-1β rs1143627G/G, rs1143643C/C, and rs16944 A/A genotypes exhibited an increased risk of cognitive decline, whereas those carrying the rs3917356C/T and T/T genotypes appeared to have a protective effect. Aluminum dust exposure appeared to modulate the above genetic susceptibility. However, this study did not find a significant association between IL-1β serum protein levels and cognitive function, suggesting that peripheral inflammation may not mediate aluminum-related cognitive decline.

Microglia are resident immune cells of the brain that remain in an inactive ‘resting’ state under physiological conditions, continuously monitoring changes in the brain microenvironment in a highly dynamic manner (Nimmerjahn et al., 2005)。When the central nervous system (CNS) is invaded by external factors, such as circulating inflammatory molecules, microglia become activated, leading to gene expression regulation, and the production of pro-inflammatory and anti-inflammatory cytokines, as well as modifications of cell surface receptors (Salter and Stevens, 2017; Yu and Ye, 2015). Chronic stimulation of microglia results in the persistent release of pro-inflammatory cytokines, such as IL-1β, IL-6, and TNF-α, which contribute to neuronal dysfunction, including impaired proliferation, differentiation, apoptosis, and synaptic plasticity (Ekdahl et al., 2009). The hippocampus, a key brain region responsible for cognition, learning, and memory, is a major site for the expression of IL-1β and its receptors (Deng et al., 2010; Jessberger and Gage, 2014). Consequently, abnormal elevations of IL-1β in the hippocampus may lead to direct impairments in memory and other cognitive functions (Zhang T. et al., 2024). In the electrolytic aluminum work environment, aluminum dust is a prevalent occupational hazard. Although aluminum is generally considered a low-toxicity metal, studies suggest that it can cross the blood-brain barrier through transferrin receptor-mediated endocytosis and transporter-mediated mechanisms (Yokel, 2001), inducing microglial activation and IL-1β expression, ultimately leading to hippocampal nerve damage (Izadi et al., 2024). Animal studies have demonstrated that increased AlCl_3_ concentrations result in microglial activation in the hippocampus, accompanied by a significant upregulation of IL-1β mRNA and protein expression levels, along with activation of downstream signaling pathways, leading to neurotoxicity (Vlas et al., 2024). This study found that cumulative exposure to aluminum dust (CED) was significantly and linearly associated with cognitive function. This provides strong epidemiological evidence for the neurotoxicity of aluminum.

Peripheral pro-inflammatory cytokines, such as IL-1β, can enter the brain through active transport by periventricular organs and by disrupting blood-brain barrier (BBB) permeability, leading to the activation of astrocytes and microglia and the initiation of a cascade of neuroinflammatory responses (Dantzer et al., 2008; Ransohoff and Brown, 2012). However, our analysis of whether peripheral IL-1β mediates cognitive decline associated with aluminum exposure revealed no significant correlation between IL-1β serum protein levels and cognitive function scores. Moreover, there was no significant difference in the serum levels of IL-1β at different cumulative exposure groups of aluminum. This finding suggests that aluminum exposure may not induce neurotoxicity through peripheral inflammatory pathways, but instead may involve other inflammatory mediators or direct neurotoxic mechanisms. Additionally, this study found that workers over 40 years of age were at a higher risk of cognitive decline. This may be attributed to the increased sensitivity of senescent microglia, which exhibit a greater propensity to release inflammatory cytokines (Norden and Godbout, 2013).

The IL-1β gene, located on human chromosome 2q14, encodes a key pro-inflammatory cytokine involved in various physiological and pathological processes. Genetic variations, particularly single nucleotide polymorphisms (SNPs), may influence gene function or expression and have been implicated in the onset and severity of multiple diseases, including tumors (Wu and Xu, 2010), Parkinson’s disease (Li et al., 2021), and coronary heart disease (Sreekanth et al., 2016). In this study, individuals carrying the rs1143627G/G, rs1143643C/C, and rs16944 A/A genotypes exhibited significantly lower cognitive function scores, suggesting that these variants may lead to increased IL-1β expression, which in turn may heighten the risk of cognitive decline. Notably, rs1143627 and rs16944 are located in the promoter region of IL-1β, and their variants may disrupt the TATA box upstream of the transcription start site, thereby altering gene expression (Xu et al., 2013; Landvik et al., 2012). rs1143643, found in an intron region, has been shown to upregulate serum IL-1β expression and increase asthma risk in children (Sobko et al., 2017). Conversely, rs3917356 C/T and T/T variants in the promoter region may reduce IL-1β expression, potentially exerting a protective effect on cognitive function. However, the functional impact of these IL-1β SNP variants varies across different diseases, and their precise role in regulating IL-1β expression remains inconclusive (Huang et al., 2024; Lawrence et al., 2024; Tian et al., 2015).

It is worth noting that the effects of IL-1β gene polymorphisms on cognitive function vary across different populations. For example, Tsai et al. reported that variation at the rs16944 locus may influence cognitive function in healthy elderly Chinese men (Tsai et al., 2010), whereas Lawrence’s study found no significant association between this variant and cognitive ability in healthy elderly American men (Lawrence et al., 2024). In the Polish population, the rs16944 variant was not associated with late-onset Alzheimer’s disease (AD) in the elderly (Klimkowicz-Mrowiec et al., 2009). However, in Canadian adolescents, this variant showed a significant association with mental disorders such as bipolar disorder (Shonibare et al., 2020). In the present study, the rs16944 variant was positively associated with cognitive decline among aluminium workers. These findings collectively suggest that the effects of IL-1β gene polymorphisms on cognitive function may differ according to population characteristics, including region, and age and so on. It is also important to highlight that most previous studies on the genetic susceptibility to cognitive impairment have focused on elderly populations. In contrast, the current study, which targeted a middle-aged and young adult occupational group, provides valuable supplementary evidence for the genetic epidemiology of cognitive impairment susceptibility.

To evaluate whether these SNP variants influence cognitive function through IL-1β serum protein levels, this study analyzed IL-1β serum levels in genotype carriers. The results indicated that carriers of rs1143627G/A, G/G, and rs16944G/A exhibited elevated IL-1β serum levels. However, only the elevated IL-1β serum level of rs1143627G/G corresponded to the cognitive decline of rs1143627G/G carriers. Despite this observation, no significant correlation was found between overall IL-1β serum protein levels and cognitive function scores. Consequently, it remains uncertain whether elevated IL-1β serum levels directly contribute to the cognitive decline linked to the rs1143627G/G variant. Furthermore, in the group with high aluminum dust exposure (CED>2.37 mg/m^3^ × year), the effect trends of rs1143627G/G, rs1143643C/C and rs16944 A/A towards decreased cognitive ability and the effect trends of rs3917356C/T and T/T towards protection were both enhanced. These findings suggest that cumulative high aluminum exposure may amplify the impact of IL-1β gene polymorphisms on cognitive susceptibility. However, no significant interaction was detected between cumulative aluminum exposure and gene polymorphisms. This lack of significance may be attributed to an insufficient sample size or the possibility that no actual interaction exists, warranting further validation in a larger cohort.

This study has several limitations. First, as a cross-sectional study, it cannot establish a clear causal relationship between aluminum exposure, IL-1β polymorphisms, and cognitive decline. Future longitudinal studies are needed to further verify this association. Second, the sample was drawn from an ethnic minority region, and geographical constraints may limit the generalizability of the findings. Third, this study found no significant association between IL-1β serum levels and cognitive function, which may indicate that the neuroinflammatory mechanism of aluminum exposure is not solely driven by IL-1β. Future research should explore other inflammatory factors to better understand the underlying mechanisms. Lastly, this study assessed cognitive function using the MoCA, which, despite its high sensitivity, may not be sufficient to detect subtle cognitive changes. Future studies should incorporate additional cognitive assessment tools to achieve a more comprehensive evaluation.

## 5 Conclusion

This study confirmed a significant linear association between occupational exposure to aluminum dust and cognitive decline. Individuals carrying the IL-1β gene rs1143627G/G, rs1143643C/C and rs16944 A/A may have a higher risk of cognitive decline. In contrast, the rs3917356C/T and T/T genotypes were negatively correlated with cognitive function scores, suggesting that they may have a protective association. Cumulative exposure to high levels of aluminum may enhance the effect of genetic susceptibility. However, further verification is required. This study did not find a significant association between IL-1β serum protein levels and cognitive function, suggesting that peripheral blood IL-1β may not be the mediating pathway for cognitive damage induced by aluminum exposure or genetic variation. These findings provide a scientific foundation for screening and intervention strategies aimed at high-risk occupational groups, with the goal of mitigating the risk of aluminum-related cognitive decline.

## Data Availability

The datasets presented in this study can be found in online repositories. The names of the repository/repositories and accession number(s) can be found in the article/Supplementary Material.
